# Encapsulation of Paraffin Phase-Change Materials within Monolithic MTMS-Based Silica Aerogels

**DOI:** 10.3390/gels9040317

**Published:** 2023-04-08

**Authors:** Linlin Xie, Xiaoxu Wu, Guichao Wang, Yury M. Shulga, Qiong Liu, Ming Li, Zhi Li

**Affiliations:** 1 School of Resources and Safety Engineering, Central South University, Changsha 410083, China; 2 Institute of Problems of Chemical Physics, Russian Academy of Sciences, 142432 Chernogolovka, Russia; 3 National University of Science and Technology MISIS, 119049 Moscow, Russia

**Keywords:** phase-change material, paraffin, MTMS-based silica aerogel, thermal management, energy storage

## Abstract

To address the leakage issue of paraffin phase-change materials in thermal management, a monolithic MTMS-based silica aerogel (MSA) is employed to encapsulate paraffin through a facile impregnation process. We find that the paraffin and MSA form a physical combination, with little interaction occurring between them. The prepared no-leakage paraffin/MSA composites have a density of 0.70 g/cm^3^ and exhibit good mechanical properties and nice hydrophobicity, with a contact angle of 122°. Furthermore, the average latent heat of the paraffin/MSA composites is found to reach up to 209.3 J/g, about 85% of the pure paraffin’s latent heat, which is significantly larger than other paraffin/silica aerogel phase-change composite materials. The thermal conductivity of the paraffin/MSA remains almost the same as that of the pure paraffin (~250 mW/m/K), without any heat transfer interference from the MSA skeletons. All these results indicate that MSA can effectively serve as a carrier material for encapsulating paraffin, which is beneficial for expanding the applications of MSAs in thermal management and energy storage.

## 1. Introduction

With energy conservation and environmental protection constantly progressing, it is significant to make efficient use of energy and recycle existing resources [1]. Obviously, developing a suitable energy-saving material is especially important [2]. It is well known to us that phase-change materials (PCMs) maintain the surrounding temperature stability by absorbing or releasing heat during the phase-change process, so as to achieve the purpose of adjusting the thermal balance [3]. PCMs control the temperature automatically by changing their shape from one form to another, which finally achieves energy recycling. Because of this characteristic, PCMs have been widely applicated in many fields, such as buildings [4], electronic components [5] and ablative materials [6].

From the literature, it is widely recognized that an acceptable PCM must meet several key points, i.e., having large latent heat, good thermal stability, high thermal conductivity, and no leakage during phase transition [7,8]. However, when reaching the phase-change temperature, the liquid–gas PCMs lead to volume expansion, resulting in difficult daily management and limiting the range of application. The solid–solid PCMs tend to have a higher phase-change temperature, which is not sensitive enough to daily temperature [9]. Hence, it is a wise choice to adopt solid–liquid PCMs to control the temperature in usual environments. According to these requirements, paraffin is an appropriate material to act as a PCM because of its inherent properties, such as being non-poisonous, possessing good thermal regulating properties and large latent heat, and having a small volume change during phase transition [10,11,12]. As we all know, paraffin is a saturated hydrocarbon with the general chemical formula C_x_H_2x+2_ [13]. It consists of linear and branched alkanes and comes from petroleum process wastes [14], making it a low-cost and easily obtainable material that conforms to the reutilization of resources.

The whole phase-transition process of paraffin can be described as follows. With the temperature rising to the melting point, the paraffin absorbs heat and slowly changes from solid to liquid; inversely, with the temperature dropping below the melting point, liquid paraffin releases heat, changing from liquid to solid. The purpose of temperature control is achieved under this phase-transition process. However, the irregular shape of liquid paraffin during the phase-change process limits its potential applications as a phase-change material [15]. An appropriate container or supporter is needed to encapsulate the liquid paraffin in case of its leakage. Various carriers have been proposed in the literature to address this issue, including microencapsulate, graphene oxide, clay minerals, nano metal powders and others [16,17,18,19]. Song et al. used a Pickering emulsion templating technique to prepare a cellulose nanofibril phase-change aerogel that had high latent heat and thermal insulation properties. However, the lower thermal conductivity limited the efficiency of heat conduction in the phase-change material [20]. Konuklu et al. utilized clay aerogel (sepiolite-based aerogel) as a carrier to absorb paraffin. The results revealed that the PCM was adsorbed in the tubular channels of sepiolite rather than on its surface, which effectively confined the paraffin in the channels to avoid leakage. However, the latent heat of the prepared composites was only 62.08 J/g, which was considerably lower than that of pure paraffin, thus compromising the phase-transition performance of the composites [21]. Cao et al. prepared graphene aerogel (GA) and GA-based paraffin phase-change composite (GAP) using the hydrothermal method. Although the GPA exhibited a high latent heat of 222.5 J/g, the photographs of GAs showed that their shape was not completely cylindrical and could not be specially customized [22]. Therefore, it is significant to find an appropriate carrier material that can prevent the leakage of paraffin and be specifically customized to its shape according to our intentions or application scenarios.

As a new porous material, silica aerogels have been paid more and more attention due to their excellent characteristics, such as high specific surface area (500–1200 m^2^/g), low density (0.003–0.500 g/cm^3^), and low thermal conductivity (17–21 mW/m/K) [23,24,25]. Based on these characteristics, silica aerogels have been widely used in many fields, e.g., acoustic application [26], aerospace application [27] and building construction [28]. Notably, silica aerogels have been proven to be effective encapsulation materials for PCMs [29]. For example, Zhou et al. used tetraethoxysilane (TEOS)-based silica aerogels to prepare the paraffin/aerogel composites and found that the heat capacity of the composites had no difference from that of the pure paraffin. However, the supporting aerogels used were powdery, which could not provide a powerful and fixed frame [30]. Li et al. encapsulated paraffin with hydrophobic and hydrophilic aerogel powders, respectively, and concluded that the hydrophilic aerogels could not prevent the leakage of paraffin [31]. Yu et al. employed monolithic TEOS-based aerogels to adsorb paraffin and obtained a phase-change composites material without leakage. However, the preparation of monolithic TEOS-based aerogels required supercritical drying, which significantly increased the costs of raw materials and time [32]. In our previous work, monolithic methyltrimethoxysilane (MTMS)-based silica aerogels (MSAs) with excellent hydrophobicity were prepared successfully under ambient pressure [33]. MSA has porous structures with micron-sized cavities [33,34,35], which can effectively prevent the generation of capillary pressure and preserve the integrity of the skeleton structure. The abundant pores in MSA can provide enough storage space for PCMs. Furthermore, MSA possesses outstanding thermostability (usually within 400–500 °C) [36] and relatively better flame retardancy [33,36,37]. Therefore, MSA should be a potential candidate for the encapsulation material of paraffin.

For the purpose of developing new PCMs, MSA is firstly synthesized by a sol-gel method and used as an encapsulation material, and then paraffin is permeated into the MSA to generate paraffin/MSA composite PCMs. The adsorption capacity of MSA is investigated in detail, as well as the physicochemical properties, microstructure, mechanical properties, hydrophobicity, and thermal properties of the prepared paraffin/MSA composites. This work demonstrates the feasibility of combining the paraffin and MSA, and the prepared paraffin/MSA composites remain effective PCMs with high latent heat and good mechanical properties. These attributes render the paraffin/MSA composites a promising candidate for thermal regulation applications, including in energy storage, building, and heating, ventilation, and air conditioning (HVAC) systems.

## 2. Results and Discussion

### 2.1. Basic Characterization of the Paraffin/MSA Composites

**Figure 1:**
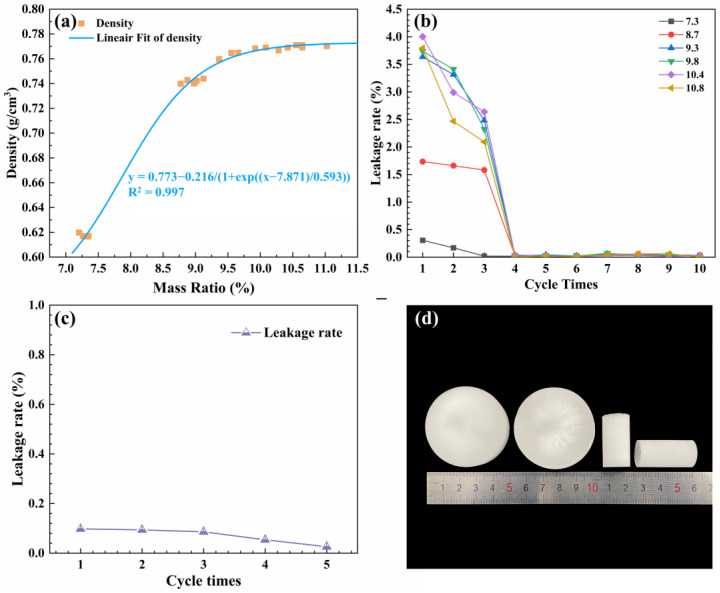
(**a**) The density versus the mass ratio, (**b**) the leakage rates at different heating–cooling cycles, (**c**) the leakage rates of the paraffin/MSA composites after three heating–cooling cycles and (**d**) photo of the samples.

As seen in Figure 1a, with the mass ratio of the paraffin to the MSA increasing from 7.2 to 10.8, the density of the paraffin/MSA composites firstly increases from 0.62 g/cm^3^ to 0.78 g/cm^3^ and then slightly oscillates around the maximum value, indicating that the MSA gradually reaches the absorption saturation of the paraffin. The presented fitting curve also implies that the density versus the mass ratio accords to a negative exponential relationship, and the maximum of the density is 0.78 g/cm^3^ without considering the occurrence of paraffin leakage. Figure 1b presents the variations in the leakage rates versus the cycle numbers. It shows that the smaller leakage rates appear during the first three heating–cooling cycles and the leakage rates dramatically drop to zero in the subsequent cycles. The initial leakages should be ascribed to the absorbed paraffin on the surface of the MSA. Once the surface paraffin is removed, no further leakage occurs, with the leakage rates reach zero. Thus, no-leakage samples are achievable by running the saturated samples through three heating–cooling cycles to remove the surface paraffin.

To better demonstrate the encapsulation capability of the paraffin/MSA composites, the leakage rates of the specimens pre-treated with three heating–cooling cycles were tested, and the results are presented in Figure 1c. It can be seen the leakage rates slightly decrease with the cycle number. All the leakage rates are less than 0.1%, which indicates that there is no leakage in practical applications. This means that the MSA provides a suitable wrapping framework for the absorbed paraffin, effectively avoiding potential leakage. From this point, we can conclude that the MSA has good encapsulation (no leakage) capacities for paraffin and the no-leakage paraffin/MSA composites are obtained through three heating–cooling cycles pre-treatment.

The density and thermal conductivity of the MSA, paraffin and no-leakage paraffin/MSA composites are shown in Table 1. The density of the paraffin/MSA is closer to that of the pure paraffin, and both are much larger than that of the pure MSA. The thermal conductivity follows a similar trend, namely, the thermal conductivity of the paraffin/MSA is much closer to that of the pure paraffin. Due to the infiltration of the liquid paraffin, the pores of the MSA are filled by paraffin, which determines the density of the paraffin/MSA composites.

As we know, effective thermal conductivity generally consists of three independent parts, i.e., solid thermal conductivity from the solid skeleton, gas thermal conductivity due to the gas molecule collision, and radiative thermal conductivity resulting from radiation interactions with the material [38]. Under room temperature, the effective thermal conductivity of MSA is determined by the solid thermal conductivity of its silica skeletons and the gas thermal conductivity in the pores. After the infiltration of paraffin, the effective thermal conductivity of the paraffin/MSA composites mainly depends on the solid thermal conductivity from the silica skeletons and paraffin. The paraffin permeating the pores of MSA provides more heat transfer channels, leading to the increase in solid thermal conductivity [39]. As a consequence, the effective thermal conductivity of the paraffin/MSA composites is much larger than that of the pure MSA. That is to say, the thermal conductivity of the paraffin/MSA composites is not reduced by the presence of MSA with low thermal conductivity but is slightly higher than that of pure paraffin. Although the thermal insulation application for the paraffin/MSA composites is no longer suitable, the higher thermal conductivity makes the composites become more sensitive to temperature changes and realize the temperature regulation function in a more timely manner, which matches the requirements of phase-change materials for high thermal conductivity [7].

### 2.2. Microstructure of the Paraffin/MSA Composites

**Figure 2:**
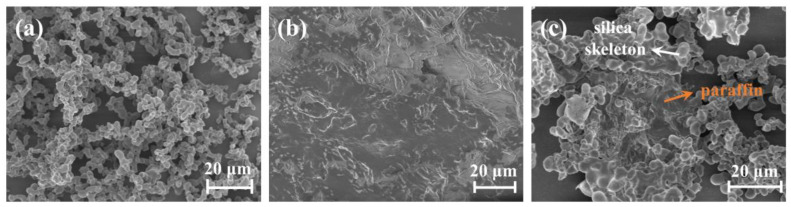
The microstructures of (**a**) the MSA, (**b**) paraffin and (**c**) paraffin/MSA composites.

Figure 2 shows the microstructures of the pure MSA, solid paraffin and paraffin/MSA composites. As shown in Figure 2a, the MSA exhibits a typical micron-scale porous silica skeleton network structure, consisting of abundant pores and coral-like branches [34,35,39]. Figure 2b reveals that the pure solid paraffin has a void-free microstructure with many wrinkles. In Figure 2c, the microstructure of the paraffin/MSA composites shows that the pores of the MSA are filled with solid paraffin, and the silica skeletons are also wrapped by the solid paraffin. Additionally, some coral-like branches from the MSA and the wrinkles from the pure solid paraffin are observed in Figure 2c.

The microstructure of the paraffin/MSA composites indicates that during the preparation, the melted liquid paraffin completely permeates into the porous silica network of the MSA. After solidification, the paraffin adheres to the silica skeletons and fills in all the pores, which finally leads to the formation of the paraffin/MSA composites. Based on the microstructure, we believe that the paraffin combines well with the silica skeletons of the MSA, and the good combination of the two confirms the feasibility and effectiveness of paraffin infiltration into MSA.

**Figure 3:**
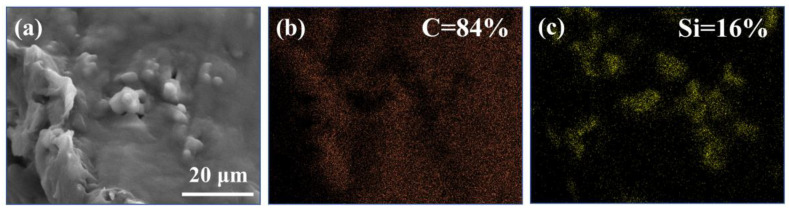
The EDS spectra of (**a**) the paraffin/MSA composites, (**b**) carbon element, (**c**) silica element.

It is known that the silicon element comes from the MSA component, and the carbon element primarily derives from the paraffin phase. The EDS spectra of the paraffin/MSA composites are present in Figure 3. It shows that the carbon element distributes evenly as a whole, while the silicon atoms distribute discontinuously, aggregating in some isolated regions. The distribution of the silicon element just accords with the structure of the porous silica skeletal network. Combining the distributions of the two elements further demonstrates that the paraffin fills the pores of the MSA during the infiltration process, and the porous silica network of the MSA provides sufficient space to accommodate the absorbed paraffin.

### 2.3. Mechanical Property of the Paraffin/MSA Composites

**Figure 4:**
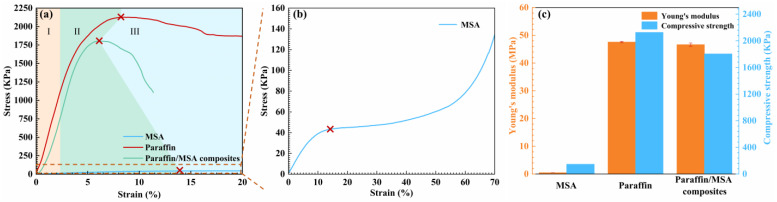
(**a**,**b**) The compressive stress–strain curves and (**c**) Young’s modulus and compressive strength of the MSA, paraffin and paraffin/MSA composites. The points labeled with red crosses represent the maximum compressive strength and the red dotted line corresponds to the enlarged stress–strain curve region.

Figure 4 shows the stress–strain curves, Young’s modulus, and compressive strength of the MSA, paraffin and paraffin/MSA composites. It can be seen from Figure 4a,b that the stress–strain curves of all materials can be divided into three stages, i.e., (I) linear elastic stage, (II) elastoplastic stage, and (III) damaged stage [34]. During stage I (ε < 3.5%), there is a good linear relationship between stress and strain, indicating that all the materials resist external forces together and can fully recover their deformation after unloading. In stage II (3.5% < ε < ε_max_), the stress increases gradually with the strain before reaching its maximum value, accompanied by the elastic and plastic deformation that occurs simultaneously. In stage III (ε > ε_max_), cracks gradually begin to appear in the materials, causing a rapid decrease in their resistance to external forces.

In Figure 4c, it is obvious that the Young’s modulus and compressive strength of the MSA are quite low, indicating that the MSA has extremely poor mechanical properties. Although the Young’s modulus of the paraffin/MSA composites is comparable to that of pure paraffin, the compressive strength is slightly reduced due to the presence of MSA. On the contrary, the Young’s modulus and compressive strength of the paraffin/MSA composites are significantly increased compared to the MSA due to the introduction of paraffin, which fills the pores of the MSA. This means that the paraffin/MSA composites still maintain good mechanical strength and are not considerably affected by the MSA. These results also demonstrate the reliability of the mechanical properties of the paraffin/MSA composites and their feasibility in practical applications.

### 2.4. Hydrophobicity and FTIR of the Paraffin/MSA Composites

**Figure 5:**
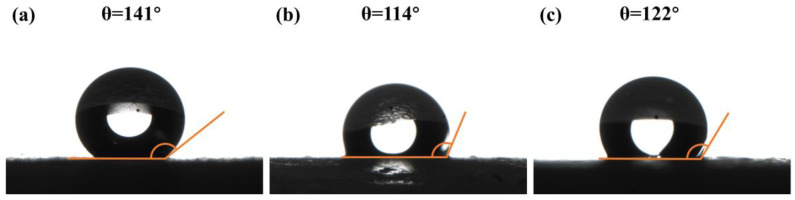
The contact angles of (**a**) the MSA, (**b**) paraffin and (**c**) paraffin/MSA composites.

In general, hydrophobicity is characterized by the water contact angle. The water contact angles of the MSA, paraffin and the paraffin/MSA composites are shown in Figure 5, respectively. The pure MSA has the largest contact angle, which is attributed to the organic groups on the silica skeletons. The pure paraffin has the smallest contact angle, but it is still greater than 90°, which is an indication of its inherent hydrophobicity. The contact angle of the paraffin/MSA composites is 122°, which is between those of MSA and paraffin, suggesting that the addition of paraffin impairs the original hydrophobicity of the MSA. In spite of that, the contact angle data of the paraffin/MSA still indicate its decent hydrophobicity, as the composites avoid a degradation of heat absorption and releasing capacity due to water infiltration.

**Figure 6:**
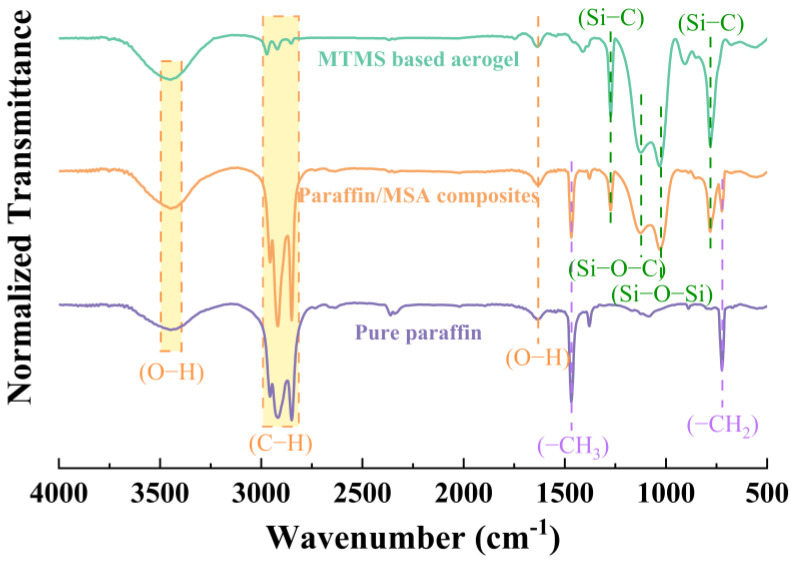
FTIR spectra of the MSA, paraffin and paraffin/MSA composites.

The FTIR spectra of the MSA, paraffin and paraffin/MSA composites are shown in Figure 6. In all three spectra, the absorption bands at 3440 cm^−1^ and 1635 cm^−1^, respectively, correspond to the stretching vibration of the O-H bonds and the bending vibrations of water molecules [40]. The absorption peaks at the wavenumbers of 2956 cm^−1^, 2890 cm^−1^ and 2848 cm^−1^ are caused by the stretching vibration of C-H bonds [30,35]. The peak at 720 cm^−1^ is due to the in-plane rocking vibration of -CH_2_ groups, which is characteristic of the crystalline state of paraffin [41]. As there is no difference between the FTIR spectra of pure paraffin and paraffin/MSA composites at 720 cm^−1^, it can be inferred that the crystalline state of paraffin remains unchanged in the composites. The bond absorption peak at 1463 cm^−1^ matches the bending vibration of -CH_3_ groups [22]. These observed hydrocarbon bonds and groups just accord with the chemical composition of paraffin, i.e., consist of linear and branched alkanes [14]. The broad band between 1126 and 1031 cm^−1^ is ascribed to the asymmetric stretching vibrations of Si-O-Si bonds [36,42], which are mainly derived from the silica skeletons of the MSA. The absorption peaks at 1274 cm^−1^ and 779 cm^−1^ are attributed to the stretching vibration and symmetric deformation vibration of Si–C bonds [39], demonstrating the existence of the Si–CH_3_ groups on the silica skeletons of the MSA [34]. The Si-CH_3_ groups constitute the chemical basis of hydrophobicity.

Comparing the IR spectra of the MSA, paraffin and paraffin/MSA composites, we find that the spectrum of the paraffin/MSA composites is the superposition of the spectra of the MSA and paraffin, and no new chemical bond is formed. This means there is no chemical reaction between the paraffin and MSA. From these IR results, we can infer that the paraffin just infiltrates into the MSA instead of reacting with it; the interfacial connection between the two is just a physical combination.

### 2.5. Thermal Analysis of the Paraffin/MSA Composites

**Figure 7:**
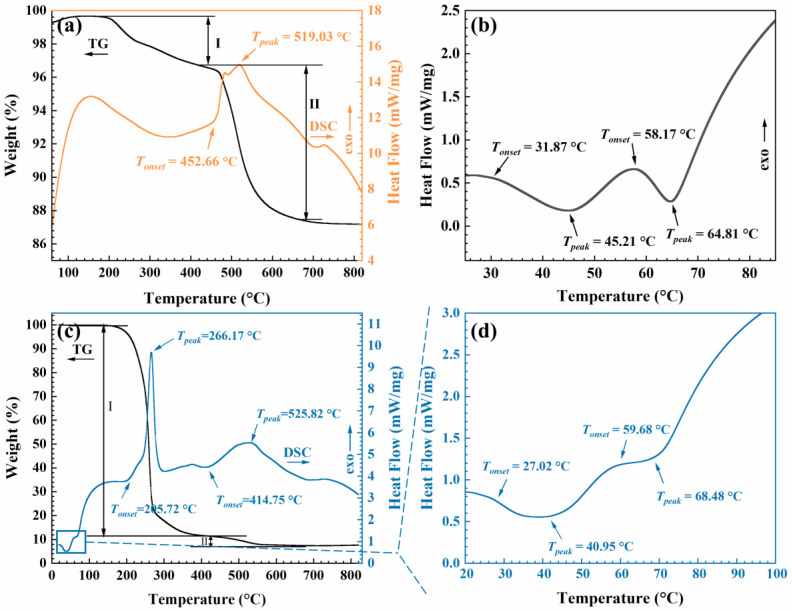
TG or DSC curves of (**a**) the MSA, (**b**) paraffin and (**c**,**d**) paraffin/MSA composites.

Figure 7 shows the TG–DSC curves of the MSA, paraffin and paraffin/MSA composites under a heating rate of 10 °C/min in an air atmosphere. As shown in Figure 7a, there are two weight-loss stages in the TG curve of the MSA. The slight weight loss in the first stage occurs from 200 °C to 420 °C, which is attributed to the residual solvent and CTAB in the MSA [39]. The primary weight loss happens in the second stage, which is caused by the thermal decomposition of the Si–CH_3_ groups on the silica skeletons of the MSA. At the same time, an exothermic peak appears around 519 °C on the DSC curve, indicating that the thermal oxidation of the Si–CH_3_ groups is an exothermic reaction [39].

As shown in Figure 7b, the paraffin has two phase-transition processes below 100 °C, which could be explained by the endothermic melting of paraffin [30]. In the first phase-transition process, the solid paraffin absorbs heat from the surrounding environment, accompanied by a simple solid-solid phase change [43], which can be interpreted as the disorder of the phase transition of monoclinic crystals to quasi-hexagonal crystals [22]. The onset temperature (*T_onset_*) is 31.87 °C and the peak temperature (*T_peak_*) is 45.21 °C. In the second phase-transition process, the paraffin changes from solid to liquid by absorbing surrounding heat, and the *T_onset_* and *T_peak_* are 58.17 °C and 64.81 °C, respectively. As we know, the high latent heat of paraffin is due to its linear straight-chain structure, which has a significant enthalpy [30]. Through calculation, the latent heat of the used paraffin is obtained as 246.1 J/g.

In Figure 7c, the weight loss is almost negligible below 200 °C, indicating that the paraffin/MSA composites have a good thermal stability, which is beneficial for their practical applications [44]. The primary weight loss of the paraffin/MSA composites can be divided into two stages. The first obvious weight loss from 205 °C to 383 °C mainly corresponds to the combustion of paraffin, with an apparent exothermic peak on DSC curve [43] and a small amount of evaporation of residual CTAB and solvent in the MSA [34]. In the first stage, the weight loss is 89%, just corresponding to the mass fraction of paraffin contained in the composites. The second weight-loss stage occurring at 415 °C is attributed to the thermal oxidation of the Si–CH_3_ groups on the MSA’s silica skeletons in the composites. The independent exothermic processes of the paraffin and Si–CH_3_ groups further verify the fact that the paraffin and MSA have only a physical combination.

In Figure 7d, the two phase-change processes observed below 100 °C belong to the absorbed paraffin in the composites. The corresponding *T_onset_* and *T_peak_* have been marked, respectively. Accordingly, the latent heat of the paraffin/MSA composites is calculated to be 209.3 J/g, which is about 85% of that of the pure paraffin. The slight reduction in latent heat is considered to be related to the interaction between the paraffin and MSA [45].

The *T_onset_* and *T_peak_* are important parameters for characterizing the thermal properties, which have been summarized in Table 2. We find that the *T_onset_* and *T_peak_* of the paraffin in the composites are almost unchanged when compared to those of the pure paraffin. Furthermore, the two DSC curves exhibit almost identical characteristics. These further indicate that the combination of the paraffin and MSA does not significantly alter the phase-transition process. The obtained paraffin/MSA composite still satisfies the thermal properties of a phase-change material.

### 2.6. Comparison between Aerogel-Based Phase-Change Materials

**Figure 8:**
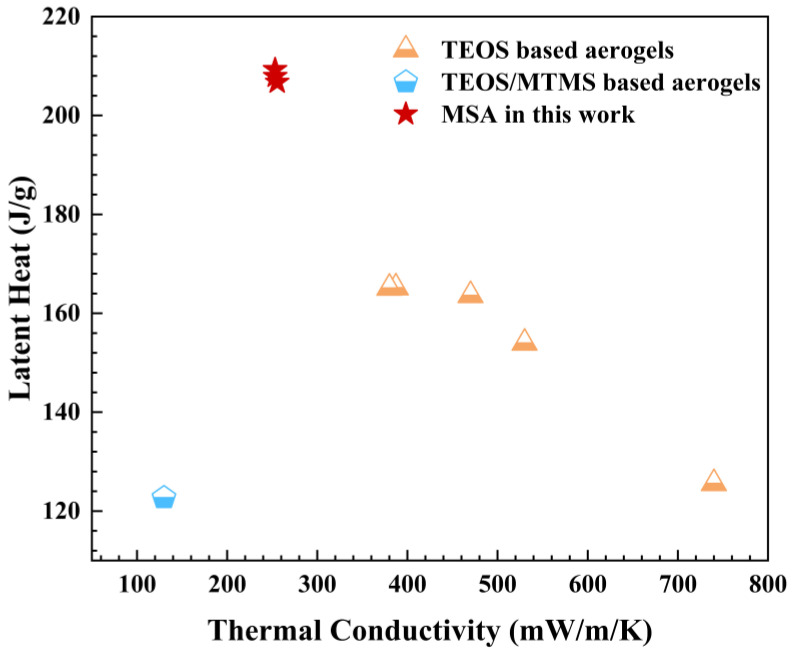
The latent heat versus thermal conductivity of paraffin/silica aerogel phase-change composite materials in this work, including TEOS-based aerogels [30,32,47], TEOS-/MTMS-based aerogels [48] and MSA.

Here, we further compare the latent heat and thermal conductivity among various phase-change materials that employ silica aerogels as carriers and paraffin as a phase-change material. As shown in Figure 8, the paraffin/silica aerogel phase-change composites mainly choose TEOS-, MTMS- and TEOS/MTMS- based silica aerogels. We note that this work is the first to use pure MSA as the sole carrier to absorb paraffin in the field of phase-change materials. Compared to other silica aerogel-based phase-change materials, the paraffin/MSA composites have the largest heat latent, which is the most significant advantage. Meanwhile, the paraffin/MSA composites exhibit a slight increase in thermal conductivity compared to pure paraffin. The related research on increasing the total thermal conductivity of the paraffin/MSA composites is in progress, which will make it possible to satisfy the high thermal conductivity requirement of phase-change materials [46]. From these perspectives, the prepared paraffin/MSA composites in this work are a potential choice for phase-change materials.

As we all know, the MSA- and TEOS-based aerogels (TSA) are two typical silica aerogels used in many application fields [35,49,50]. Here, we present the physical parameters of these two silica aerogel-based phase-change materials in Table 3. As is widely reported, TSA is usually obtained as powders and fragments, and the prepared aerogel-based phase-change materials using TSA as carriers are also in the same form [30,47,51]. In this work, we easily prepare monolithic MSA and subsequently use the MSA to absorb paraffin to generate paraffin/MSA composites. Obviously, the monolithic paraffin/MSA has a larger advantage in thermal management applications, providing a stable framework for absorbed paraffin and avoiding potential leakage. For comparing the preparation of MSA and TSA, MSA can be prepared in pure water within a relatively shorter period (~12 h), while several organic solvents are needed for the synthesis of TSA and the preparation period is far longer (>72 h). The obtained TSA has a typical nanoporous structure, while MSA has a microstructure with micron-sized cavities [33,34,35]. Moreover, the pore volume of MSA is much larger than that of TSA. As a consequence, the paraffin absorbed by MSA is much larger than that absorbed by TSA, which maintains a larger latent heat and has better thermal regulation properties.

## 3. Conclusions

In this study, monolithic and high-latent-heat paraffin/MSA composites with no leakage were successfully fabricated through a simple infiltration process. This demonstrates that the paraffin fills the porous structure of the MSA, resulting in a physical combination between the two components. The obtained paraffin/MSA composites possess the largest latent heat among all known paraffin/silica aerogel phase-change composite materials. Importantly, to our best knowledge, this work reports the use of MSA as the sole carrier for encapsulating paraffin and preparing phase-change composite materials for the first time. Taken together, our findings highlight the feasibility of using MSA as a carrier material to encapsulate paraffin, which has the potential to further advance the development of MSA-based phase-change materials and broaden their applications in thermal management and energy storage.

## 4. Experimental Section

### 4.1. Raw Materials

The methyltrimethoxysilane (MTMS, 98%), paraffin (C_x_H_2x+2_, melting point: 54~56 °C, CAS: 8002-74-2) and nitric acid used in the experiments were from Aladdin Reagent Co., Ltd. (Shanghai, China). Cetyltrimethylammonium bromide (CTAB, 99%) and ammonium hydroxide (NH_3_∙H_2_O, 25~28%) were purchased from Sinopharm Chemical Reagent Co., Ltd. (Shanghai, China). All these chemicals were used as received without further purification. Deionized water was produced by a laboratory water purification system (Eco-S15UVFV, HHitech, Shanghai, China). and was used to prepare 0.1 M HNO_3_ (aq) and 1 M NH_3_∙H_2_O (aq), respectively.

### 4.2. Sample Preparation

**Figure 9:**
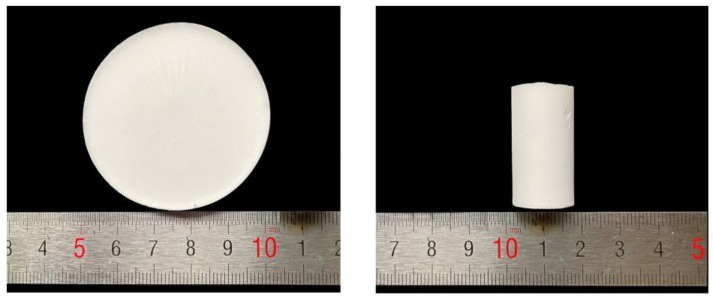
The pie-like and cylindrical MSAs.

Preparation of MSA: Firstly, MTMS, deionized water, CTAB and 0.1 M HNO_3_ were mixed together in a beaker and kept stirring for 3 min. After sufficient hydrolysis in a 45 °C water bath for 30 min, 1 M NH_3_·H_2_O was added to the mixed solution, and then the hybrid solution was kept stirring for 10 s. The obtained mixture was poured into cylindrical glass molds (Φ15.5 mm × 30 mm) and pie-like silicone molds (Φ48 mm × 8 mm), waiting for gelation. After the gels were formed, the wet gels were aged for 7–8 h, then the wet gels were dried under ambient pressure at 100 °C for 4 h to generate monolithic MSA. The obtained pie-like and cylindrical MSAs are presented in Figure 9.

Preparation of paraffin/MSA composites: The beaker containing weighed paraffin was heated in an oven at 100 °C until the paraffin completely melted. Then, the MSA was added to the beaker to absorb the liquid paraffin. The MSA was kept in the liquid paraffin for over 10 min to ensure the full immersion of the paraffin. Subsequently, the MSA with the liquid paraffin was taken out and cooled for solidification, to obtain the paraffin/MSA composites. In this work, the mass ratio of the initially weighed paraffin to the MSA ranged from 7.2 to 10.8, to investigate whether the paraffin/MSA composites were saturated for paraffin absorption.

Preparation of no-leakage paraffin/MSA composites: In order to prevent the paraffin from leaking during the heating process, all prepared paraffin/MSA composites were pretreated with three heating–cooling cycles, where one cycle corresponded to heating in an oven at 100 °C for 30 min and cooling at room temperature for 30 min. These operations were aimed at removing residual paraffin from the surface of the MSA, which is exhibited in Section 2.1.

### 4.3. Methods of Characterization

The leakage rate was determined by the following formula,

(1) $$\mathrm{Leakage}\;\mathrm{rate}\;\left(\%\right)=\left(1-\frac{m_{1}}{m_{0}}\right)\times 100\%$$

in which the m_0_ and m_1_ denote the initial mass and the remaining mass before and after one heating–cooling cycle, respectively. It should be noticed that only the specimens without leakage were used for the tests below.

The densities of the pure MSA, paraffin and paraffin/MSA composites were calculated from the measured mass and volume. The microstructures were observed by a field emission scanning electron microscope (SEM, ZEISS Sigma 300, Oberkochen, Germany), accompanied by energy dispersive spectroscopy (EDS, EDX-720, Shimadzu Corporation, Kyoto, Japan). The mechanical properties of the cylindrical samples were tested by a uniaxial compression test (MST Insight 30, Minneapolis, MN, USA) with a loading rate of 1 mm/min. The chemical bonds and groups of the specimens were studied by Fourier transform infrared spectroscopy (FTIR, Nicolet 8700, Nicolet, Madison, WI, USA) via a KBr pellet method, within a wavenumber from 400 to 4000 cm^−1^. The thermal conductivity was measured with a thermal constant analyzer (XIATECH, TC3000E, Xian, China) under ambient temperature and pressure. The hydrophobicity was evaluated by a contact angle instrument (JC2000D1, Shanghai Zhongchen Instrument, Shanghai, China). In the measurement, a 5 μL water drop was added on the surface of the specimen, and the image of the water drop on the surface of the sample was obtained by a high-definition camera. The contact angle was calculated through an image analysis software [52]. The thermal properties of the specimens were investigated by TG-DSC (SDT Q600, TA Instrument, New Castle, NH, USA) with a heating rate of 10 °C/min, and the latent heat was calculated based on the DSC curves.

## Figures and Tables

**Table 1:** Physical properties of the MSA, paraffin and paraffin/MSA composites.

Physicochemical Property	MSA	Paraffin	Paraffin/MSA Composites
Density (g/cm^3^)	0.074	0.82	0.70
Thermal conductivity (mW/m/K)	30.9	246.3	253.1

**Table 2:** Thermal parameters of different thermal processes.

Parameters	Paraffin in the Composites (<80 °C)	Paraffin	Paraffin/MSA Composites	MSA
I-*T_onset_* (°C)	27.02	31.87	205.72	-
I-*T_peak_* (°C)	40.95	45.21	266.17	-
II-*T_onset_* (°C)	59.68	58.17	414.75	452.66
II-*T_peak_* (°C)	68.48	64.81	525.82	519.03

**Table 3:** Physical properties of two typical paraffin/silica aerogel phase-change composites.

Properties	Paraffin/MSA	Paraffin/TSA [30,47,51]
shape of aerogels	monolithic	powdery
solvent for aerogel preparation	pure water	multiple organic solvents
preparation time (h)	~12	>72
pore size of aerogels	micron-	nano-
mass ratio of paraffin to aerogels	>8	3

## Data Availability

Not applicable.
